# Die Zügeloperation bei inkompletter Okulomotoriusparalyse (Rectus-superior- und Rectus-lateralis-Transposition nach nasal unten)

**DOI:** 10.1007/s00347-021-01339-y

**Published:** 2021-03-01

**Authors:** Michael Gräf

**Affiliations:** Klinik und Poliklinik für Augenheilkunde, Universitätsklinikum Gießen und Marburg GmbH, Standort Gießen, Friedrichstr. 18, 35392 Gießen, Deutschland

**Keywords:** Augenmuskelchirurgie, Okulomotoriusparese, Paralytisches Schielen, Transpositionsoperation, Nasale Transposition, Extraocular muscle surgery, Strap operation, Oculomotor nerve palsy, Paralytic strabismus, Nasal transposition

## Abstract

**Ziel:**

Demonstration der Technik und Wirksamkeit der Zügeloperation bei Okulomotoriusparalyse mit erhaltener Hebungsfähigkeit.

**Methode:**

Transposition der Mm. recti lateralis (hinter Mm. obliquus und rectus inferior) und superior (hinter Obliquus-superior-Sehne und M. rectus medialis) in den nasal unteren Quadranten.

**Ergebnisse:**

Im exemplarisch dargestellten Fall korrigierte der Eingriff ohne wesentlichen Verrollungseffekt eine paralytische Exo-Hypertropie von 30–40°/20–30°.

**Schlussfolgerung:**

Die Zügeloperation erweitert das chirurgische Repertoire zur Korrektur paralytischen Schielens.

## Abstract (en)

**Purpose:**

To demonstrate the technique and effectiveness of the bridle operation for oculomotor nerve paralysis with maintained elevation.

**Method:**

The bridle operation includes transposition of the lateral rectus muscle (passing behind inferior oblique and rectus muscles) and the superior rectus muscle (behind superior oblique tendon and medial rectus muscle) to the nasal inferior surface of the globe.

**Results:**

In the reported case, this procedure corrected exo-hypertropia of 30–40°/20–30° without inducing cyclotropia.

**Conclusion:**

The bridle operation expands the surgical arsenal for paralytic strabismus.

Im Jahr 1990 stellte Kaufmann in dieser Zeitschrift (damals *Fortschritte der Ophthalmologie*) eine spektakulär anmutende Transpositionstechnik vor, die sog. Zügeloperation [8]. Wenig später, im Jahr 1991, beschrieb er das „Lateralis-Splitting“ zur Stellungskorrektur bei kompletter Okulomotoriusparalyse [9]. Die Indikation zum Lateralissplitting, das erst 2 Jahrzehnte nach seiner Einführung international breite, an publizierten Kasuistiken und ersten kleinen Fallserien erkennbare Aufmerksamkeit erlangte, ist häufiger gegeben als zur Zügeloperation, die bis heute außerhalb des deutschen Sprachraums weithin unbekannt und in der englischsprachigen Fachliteratur kaum erwähnt ist [18]. Die Zügeloperation dient zur Korrektur von Schielstellungen durch den Ausfall zweier gerader Nachbarmuskeln bei vorhandener Funktion der beiden anderen Recti. Diese Konstellation kann bei einer isolierten Läsion des unteren Okulomotoriusastes in der Orbita auftreten, welcher die Mm. recti inferior und medialis, den M. obliquus inferior und das Ganglion ciliare innerviert, oder z. B. im Rahmen einer nukleären Okulomotoriusparalyse, die den M. rectus superior ausspart, weil dessen Innervation vom kontralateralen Kern her erfolgt [4, 6, 13, 19]. Das Hebungsdefizit ist deswegen geringer als auf der anderen Seite, und das Auge steht weit exo- und hypertrop. Die Ptosis ist eher symmetrisch [14]. Eine dauerhafte Stellungskorrektur ist bei paralytischem Schielen schwierig und in vielen Fällen nur durch Muskeltranspositionen möglich. Isolierte Rücklagerungen, Verkürzungen und kombinierte Rücklagerungs- und Verkürzungseingriffe sind weniger Erfolg versprechend. Aufgrund ihrer funktionellen Integrität sind in diesem Fall die ipsilateralen Mm. recti superior und lateralis geeignete Kandidaten für eine Transposition.

## Entwicklung der Zügeloperation

**Figure 1:**
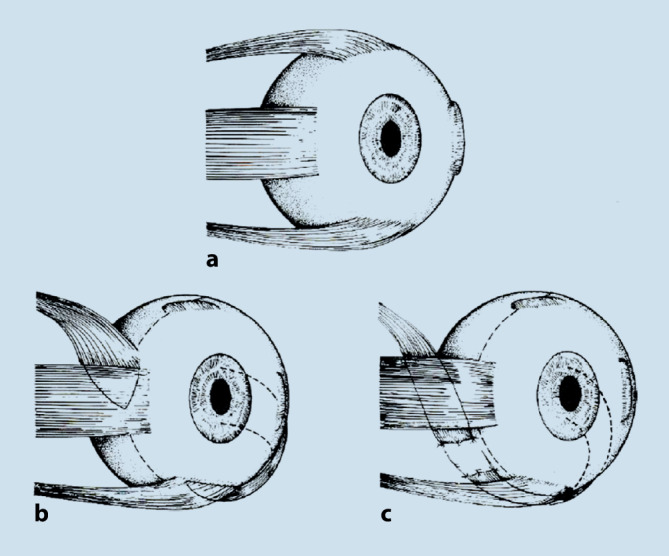

In der Originalarbeit wird über 2 Patienten mit Paralyse der Mm. recti inferior und medialis mit Beteiligung der inneren Augenmuskeln berichtet [8]. Im ersten Fall, einer 46-jährigen Patientin mit rechtsseitiger Okulomotoriusparese nach einem zerebrovaskulären Insult, betrug die Exotropie 30° und die Hypertropie 20° ohne Zyklodeviation. In Vollnarkose wurde der M. rectus superior unter der Obliquussehne hindurch zur Insertion des M. rectus medialis verlagert, kombiniert mit einer retroäquatorialen Myopexie 14 mm hinter der neuen Insertion, um dem Muskel ein senkendes Drehmoment zu geben. Analog dazu wurde der M. rectus lateralis hinter dem M. obliquus inferior hindurch an die Insertion des M. rectus inferior angelagert und ebenfalls mit einer hinteren Fixationsnaht versehen, also ähnlich einer Fadenoperation nach Cüppers [3], jedoch zu einem anderen Zweck. Der Eingriff verminderte die Exotropie um 30°, die Hypertropie um fast 20° und erzeugte nur 4° Exzyklodeviation, was der Patientin binokulares Einfachsehen im Geradeausblick ermöglichte [8]. Der zweite Patient, 65 Jahre alt, hatte 1942 eine Kopfverletzung erlitten mit der Folge einer absoluten Pupillenstarre des linken Auges. Erst ab 1950 wäre Diplopie aufgetreten, die ab 1970 auch bei extremer Kopffehlhaltung nicht mehr verschwand. Deshalb und aus ästhetischen Gründen wünschte der Patient eine Operation. Im Geradeausblick betrug die Exotropie 50°, die Hypertropie 20°, eine Zyklodeviation bestand nicht. In einem geplant schrittweisen Vorgehen erfolgten zunächst die Transpositionen wie beschrieben, jedoch ohne Myopexie, was die Exo- und Hypertropie nur um je 15° verringerte. Vier Wochen später wurden die transponierten Muskeln an ihren neuen Insertionen abgetrennt, durch Fascia lata verbunden und diese im nasal unteren Quadranten 25 mm vom Hornhautrand entfernt an der Sklera fixiert (Abb. 1). Daher rührt die Bezeichnung Zügeloperation [8]. Das ca. 6 mm lange Transplantat sollte einer Überspannung der durch die permanente Fehlstellung verkürzten Muskeln mit einer evtl. Gefäßkompression vorbeugen (H. Kaufmann, persönliche Mitteilung). Der Gesamteffekt beider Eingriffe betrug 30° in horizontaler und 20° in vertikaler Richtung [8]. Eine Zyklotropie bestand auch postoperativ nicht. Bei 5° Kopfrechtsdrehung verfügte der Patient über Einfachsehen mit Fusion [8].

In der Folge ohne Myopexie und ohne Implantat [10–12, 18] wurde die solchermaßen zügellose Zügeloperation eine wertvolle Ergänzung unseres chirurgischen Repertoires. Ausfallmuster, bei denen sie in Betracht kommt, sind relativ selten. Eine exemplarische Beschreibung der letztgenannten Variante erläutert das operative Vorgehen.

## Aktuelles Fallbeispiel

**Figure 2:**
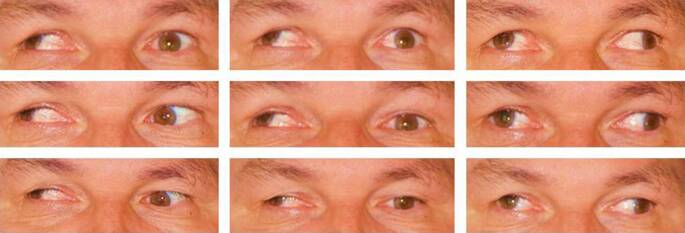

**Figure 3:**
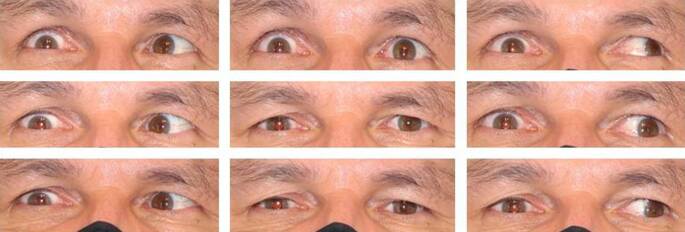

Der 55-jährige Patient litt seit 2 Jahren nach ätiologisch ungeklärten ischämischen Insulten im Bereich des posterioren Thalamus und des Mesenzephalons an einer Hemiparese links, einer vertikalen Blickparese und einer rechts stärker als links ausgeprägten inkompletten Okulomotoriusparese mit entsprechender Diplopie (Abb. 2). Die Hebung war am RA auf 10–15°, am LA auf 5–10° eingeschränkt, die Senkung auf 5° bzw. 15°, die Adduktion war am RA nur bis 15° vor die Mittellinie, am LA 40° weit möglich. Die Abduktion war beidseits frei. Wegen des Motilitätsdefizits nahm der Patient bei Rechtsfixation 40° Kopflinksdrehung ein, bei Linksfixation 10° Rechtsdrehung und 10° Hebung. Gemessen mit beidseits vorgehaltenen, in Grad kalibrierten Prismen stand das RA nach Lage der Hornhautspiegelbilder 47° (in der Nähe 52°) exo- und 20° hypertrop. Angaben zur Zyklodeviation waren nicht zu erhalten. Die Inzykloduktion des RA im versuchten Abblick zeigte eine erhaltene Funktion des M. obliquus superior. Somit bestand keine wesentliche Trochlearisparese. Zunächst sollte die sehr auffällige Stellungsabweichung des stärker paretischen RA korrigiert werden, das in seiner Position funktionell kaum nutzbar war. Der Patient wurde informiert, dass auf Wunsch ein zweiter, kleinerer Eingriff (eine kombinierte Divergenzoperation) am LA zur Verbesserung der Kopfhaltung und eines evtl. restlichen Außenschielens möglich wäre. Die Operation erfolgte in Vollnarkose über einen temporalen Türflügelschnitt, der einen übersichtlichen Zugang schafft, und eine radiäre, auf die Sklera durchgreifende Inzision nasal unten. Der M. rectus lateralis wurde am oberen und unteren Drittel mit doppelt armiertem 6‑0 Polyglactin angeschlungen, von der Sklera abgetrennt und an den Fäden zuerst hinter dem dorsal großstreckig freipräparierten M. obliquus inferior durchgezogen. Anschließend wurden die Fäden mit einem Barraquer-Nadelhalter, dicht auf der Sklera bleibend, unter dem M. rectus inferior zur nasalen Öffnung durchgereicht und dort mit einer Pinzette gefasst. Nachdem zunächst die Fäden und an diesen der M. rectus lateralis nach nasal gezogen waren, wurde der Muskel mit dem Vorderrand 15 mm vom Limbus entfernt provisorisch an der Sklera fixiert. Die Hypertropie war dadurch kaum verringert. Nach Verlängerung des Limbusschnitts wurde der M. rectus superior entsprechend mit 6‑0 Polyglactin angeschlungen, abgetrennt und mit dem Nadelhalter hinter der Sehne des M. obliquus superior (auf ein Häkchen oder Silikonbändchen genommen; keine Fasern dorsal des Transponats, wie zuvor am Obliquus inferior) unter dem M. rectus medialis hindurch zur nasalen Öffnung geführt und dicht über der dort befindlichen Lateralissehne verankert. Das Auge stand nun gering gesenkt. Daraufhin wurde die Lateralisnaht endgültig fixiert und die Bindehaut mit 9‑0 Polyglactin verschlossen. Bei Entlassung bestand 5° (in der Nähe 11°) Exotropie mit 6° (in der Nähe 10°) Hypotropie und 6° Exzyklotropie, die im geringen Abblick auf 0° abnahm. Mit einem Prisma von 10° Basis oben war punktuell binokulares Einfachsehen in 5° Abblick nachweisbar. Die Heilung verlief problemlos. Eine seröse Amotio, wie öfter nach Lateralissplitting [1], trat nicht auf. Nach 6 Monaten war die Augenstellung unverändert. Der Visus betrug wie vor der Operation am RA 0,5, am LA 1,25. Der Patient hatte keine Missempfindungen. Mit dem ästhetischen Resultat war er zufrieden (Abb. 3). Die Diplopie störte nicht stärker als vor dem Eingriff, jedoch weiterhin, sodass er die meiste Zeit eine monokulare Okklusion der Prismenfolie vorzog.

## Wirkung der Zügeloperation

**Figure 4:**
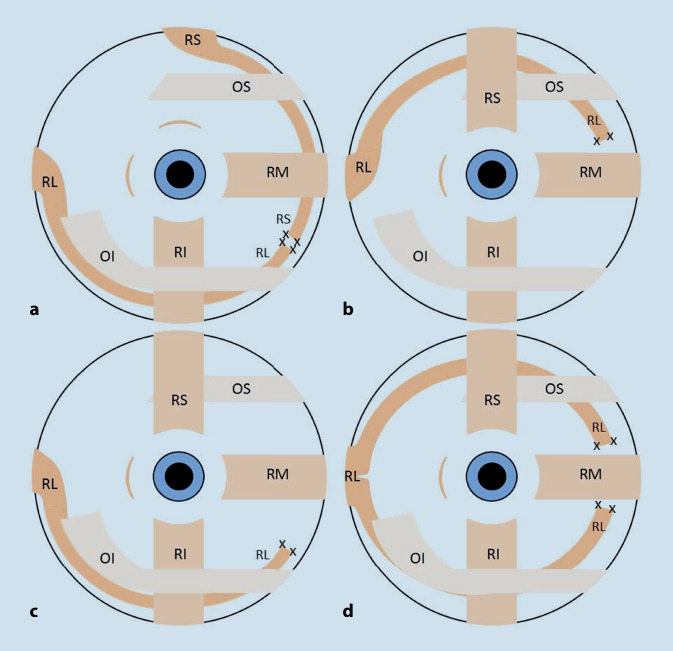

Die nicht paretischen Recti, die das Auge mit oder ohne Obliquusbeteiligung in die Schielstellung ziehen, entfalten nach ihrer Transposition ein horizontales und vertikales, in der Vektorsumme dem Schielen entgegengerichtetes Drehmoment. Da sich ihre zyklorotatorischen Kräfte neutralisieren, entsteht keine Verrollung. Der adduzierende Effekt geht vorwiegend vom M. rectus lateralis aus, der gleichzeitig senkt und einwärts rollt [5]. Der transponierte M. rectus superior hat im Wesentlichen zyklovertikale Wirkung. Um eine Oberlidverformung zu vermeiden, wird er hinter der Obliquussehne durchgeführt (Abb. 4). Diese ist selbst im Fall einer Trochlearisparese für weitere Maßnahmen verfügbar, anders als nach einer Tenektomie [13, 16]. Von einer Tenotomie oder Myektomie des paralytischen Obliquus inferior ist kein Nachteil zu erwarten.

## Vorstufen und Alternativen

Für vergleichbare klinische Situationen nannte Knapp 1989 als Therapieoption eine Verlagerung derselben Muskeln, die an den Nachbarmuskeln endete [15]. Der M. rectus lateralis wurde neben die Insertion des M. rectus inferior verlagert, der M. rectus superior über die Insertion des M. rectus medialis. Knapp hatte diese Transposition seit 1964 mehrfach vorgenommen und bereits 1978 beschrieben, dass sie die Schielstellung deutlich verringerte [13, 15]. Später erzielte Kushner damit bei 5 Patienten mit moderater Exo-Hypertropie von durchschnittlich 36 bzw. 22 PD eine Verbesserung auf durchschnittlich 5,5 bzw. 4 PD [16]. Am Goldmann-Perimeter (also in der Nähe und ohne Kontrolle auf Zyklotropie) war ein diplopiefreier Blickbereich nachweisbar [16]. Ebenfalls 1989 beschrieb Taylor eine Patientin, deren paretisches Auge 40° exo- und 15° hypotrop stand. Er verlagerte den M. rectus lateralis unter dem M. rectus superior hindurch nach nasal und fixierte ihn zwischen den Mm. recti medialis und superior ca. 11 mm vom Hornhautrand entfernt [20]. Die Exotropie wurde dadurch auf 15° reduziert, die Hypotropie kaum. Weitere Eingriffe folgten. Durch diese Transposition auf dem oberen Weg bekommt der M. rectus lateralis neben der adduzierenden eine hebende und stark exzykloduzierende Wirkung (Abb. 4b). Trotz intakter Funktion des M. obliquus superior entsteht eine Exzyklotropie. Bei Transposition auf dem unteren Weg (Abb. 4c) erhält er adduzierende, senkende und stark inzykloduzierende Wirkung [5, 17, 20]. Diese Einmuskeltranspositionen können für eine ästhetisch befriedigende Stellungskorrektur genügen [5]. Wenn die dadurch induzierte Zyklotropie besonders stört, ist die Umwandlung zur Zügeloperation leicht möglich. Darauf im Aufklärungsgespräch hinzuweisen ist unbedingt wichtig. Ebenfalls wichtig ist vor einer Zügeloperation der Hinweis auf einen möglichen Übereffekt, der eine Lockerung der Transponate erfordert.

Die Zügeloperation vereint die Vorteile der Methoden von Knapp und Taylor. Durch die Verwendung von beiden nicht paretischen Muskeln induziert sie keine Verrollung, und durch die Transposition auf die jeweils andere Bulbushemisphäre wird ihr Zug in die Gegenrichtung umgelenkt. Die neue Zugrichtung entsteht erst innerhalb der Tenonkapsel (Abb. 4). Die proximale Muskelzugrichtung vom Anulus tendineus communis zur Tenonpforte ändert sich kaum. Die Tenonpforte ist für die orbitale Muskelschicht ein Insertionsort und fungiert durch ihre elastische Aufhängung an der Periorbita für die bulbäre Muskelschicht als Hypomochlion („Pulley“). Diese Funktion erklärt die Wirkung von Transpositionen, die dadurch definiert sind, dass ein Muskel aus seiner eigentlichen Zugrichtung verlagert wird und damit eine andere Zugrichtung erhält. Das beschränkt sich im Wesentlichen auf den Verlauf des kurzen distalen Muskelsegments im subtenonalen Raum zwischen Tenonkapsel und Sklera. Wie jede konventionelle Augenmuskeloperation sind Transpositionen in diesem Sinn intrakapsuläre Eingriffe. Das ist auch bei kleineren, z. B. vertikalen Verlagerungen von Horizontalmotoren besonders im Hinblick auf torsionale Effekte zu bedenken. Beim Kind mit sehr stabilem Bindegewebe kann der Transpositionseffekt stärker sein als im höheren Lebensalter, in dem die Bindegewebestrukturen nachgiebiger sind.

Abschließend sollte erwähnt werden, dass auch der adduzierende Effekt des Lateralissplittings (Abb. 4d) erst durch die Unterkreuzung der vertikalen Recti entsteht [1, 9, 11]. Die Wirkung der Hummelsheim-Operation zur Behandlung bei Abduzensparalyse kann nach Vorschlag von Kaufmann ebenfalls durch primäres Unterkreuzen des Nachbarmuskels, in diesem Fall des M. rectus lateralis, verstärkt werden [7]. Diese Kreuzung erhöht wirkungsvoller als die von O’Connor vorgestellte Variante die Spannung der Transponate, ändert aber nicht grundlegend ihre Zugrichtung.

## Fazit für die Praxis

Die Zügeloperation ermöglicht bei Paralyse zweier benachbarter Recti eine Normalisierung der Physiognomie und eine Reduktion des Doppelbildabstands und der binokularen Konfusion. Binokulares Einfachsehen ist aufgrund der Paralyse nur in einem sehr kleinen Blickrichtungsareal erreichbar, in allen anderen Richtungen besteht Diplopie. Bei Blickbewegungen verschiebt sich das Doppelbild in die jeweilige Blickrichtung, was auf andere Weise beeinträchtigt als die Diplopie und Konfusion im zuvor großen Schielwinkel. Darüber muss der Patient vor dem Eingriff aufgeklärt werden. Zudem interferiert der optokinetische Reflex, den das bewegte Netzhautbild des paretischen Auges bei Kopfbewegungen auslöst, mit der übrigen Blickmotorik. Daher benötigen Patienten eventuell auch postoperativ eine Brillen- oder Kontaktlinsenokklusion. Abgesehen von der Unterbrechung der Muskelgefäße ist die Zügeloperation kurzfristig komplett reversibel. Sie scheint in Händen operativ erfahrener Strabologen kein wesentliches Risiko für die Sehfunktion des Auges zu bergen. Eine präzisere Aussage ist anhand der geringen Fallzahlen nicht möglich.

## References

[1] Basiakos S, Gräf M, Preising M, Lorenz B (2019). Splitting of the lateral rectus muscle with medial transposition to treat oculomotor palsy: a retrospective analysis of 29 cases. Graefes Arch Clin Exp Ophthalmol.

[2] Chaudhuri Z, Demer JL (2015). Magnetic resonance imaging of bilateral lateral rectus split transposition to the medial globe. Graefes Arch Clin Exp Ophthalmol.

[3] Cüppers C, Fells P (1976). The so-called “Fadenoperation” (surgical correction by well-defined changes in the arc of contact). Transactions of the 2nd Congress International Strabismological Association 1974.

[4] Cunningham ET, Good WV (1994). Inferior branch oculomotor nerve palsy: a rare case report. J Neuroophthalmol.

[5] Gräf M, Lorenz B (2010). Nasal-inferiore Transposition des M. rectus lateralis bei Okulomotoriusparese. Klin Monbl Augenheilkd.

[6] Ing E, Sullivan T, Clarke M, Buncic J (1992). Oculomotor nerve palsies in children. J Pediatr Ophthalmol Strabismus.

[7] Kaufmann H, Kaufmann H (1986). Augenmuskeloperationen. Strabismus.

[8] Kaufmann H (1990). Die sogenannte Zügeloperation. Fortschr Ophthalmol.

[9] Kaufmann H (1991). „Lateralissplitting“ bei totaler Okulomotoriusparalyse mit Trochlearisparese. Fortschr Ophthalmol.

[10] Kaufmann H, Kaufmann H (1995). Paretisches Schielen. Strabismus.

[11] Kaufmann H, Gómez de Liaño R (2007). Surgical procedures in the treatment of paralytic strabismus. Transactions 31st Meeting European Strabismological Association, Mykonos.

[12] Kaufmann H (2011). Operative Versorgung der Okulomotoriusparese. Z Prakt Augenheilkd.

[13] Knapp P (1978). Paretic squints. Symposium on Strabismus. Transactions of the New Orleans Academy of Ophthalmology.

[14] Kommerell G, Lagrèze WA, Steffen H, Kaufmann H (2020). Neurogene Augenmuskellähmungen. Strabismus.

[15] Kushner B (1989). Grand rounds #15: a case of paresis of the inferior division of the IIIrd nerve. Binocul Vis Strabismus Q.

[16] Kushner B (1999). Surgical treatment of paralysis of the inferior division of the oculomotor nerve. Arch Ophthalmol.

[17] Morad Y, Nemet P (2000). Medial transposition of the lateral rectus muscle in combined third and fourth nerve palsy. J AAPOS.

[18] Roth A, Speeg-Schatz C (2001). Eye muscle surgery. Basic data, operative techniques, surgical strategy.

[19] Susac J, Hoyt W (1977). Inferior branch palsy of the oculomotor nerve. Ann Neurol.

[20] Taylor JN (1989). Surgical management of oculomotor nerve palsy with lateral rectus transplantation to the medial side of the globe. Aust N Z J Ophthalmol.

